# Education disrupts the intergenerational transmission of health disadvantage across three generations in China

**DOI:** 10.1371/journal.pone.0302963

**Published:** 2024-06-07

**Authors:** Weijuan Wu, Haokai Liao, Xuelin Yang

**Affiliations:** 1 School of Economics and Management, South China Normal University, Panyu District, Guangzhou City, Guangdong Province, China; 2 College of Humanities and Arts, Heyuan Polytechnic, Yuancheng District, Heyuan City, Guangdong Province, China; 3 The School of Marxism, Jiangxi University of Technology, Gaoxin District, Nanchang City, Jiangxi Province, China; University of Jyvaskyla, FINLAND

## Abstract

This article utilizes survey data from the China Family Panel Studies (CFPS) to examine whether grandparents’ health disadvantage have both direct and indirect effects on the health disadvantage of their grandchildren, and whether the completion of compulsory education by parents disrupts these intergenerational transmissions in China. The findings suggest that grandparents’ health disadvantage significantly increases the probability of grandchildren’s health disadvantage with and without controlling parental health disadvantage and other characteristics. Moreover, the study identifies a disruptive influence of parental education on this transmission process. Rigorous robustness tests, including the use of the Compulsory Education Law as an instrumental variable to control for unobserved factors, validate these results. Mechanism analysis shows that parents completing compulsory education contribute to improving their nutritional balance and adopting healthy behaviors, attaining higher social status, earning higher income, which ultimately reduce the probability of health disadvantage for both themselves and their children. These findings highlight the persistent intergenerational transmission of health disparities within families and emphasize the importance of enhancing individuals’ education levels to disrupt this transmission. By doing so, it may be possible to mitigate health inequalities and disparities across the population.

## Introduction

Health is an important component of human capital. From an individual perspective, good health plays a vital role in improving educational attainment and fostering the development of social capital, which in turn can influence employment status, types of employment, and ultimately impact income and social class [1]. From a family perspective, good health can increase household income, reduce medical expenses, and promote family happiness [2]. From a socio-economic perspective, good health is beneficial for promoting labor supply, extending individuals’ working years, improving labor productivity, and ultimately driving economic growth [3].

In China, substantial advancements in socioeconomic progress and healthcare have led to overall enhancements in population health. Data from the 2022 China Statistical Yearbook [4], spanning from 1981 to 2020, reveals an increase in average life expectancy from 67.77 years to 77.93 years. It does not mean everyone can benefit equally, as there are disparities in health among different social and economic statuses, gender, regions, and healthcare levels [5]. Disparities in health are also evident across different educational levels, as highlighted by the 2013 National Health Service Survey [6].

An individual’s health level is influenced by genetic factors [7–9], as well as familial socioeconomic status, healthy behaviors, and access to health education [10,11]. Among these factors, the influence from the family is the most important. Numerous studies have underscored the presence of intergenerational transmission of health within families [12–14]. In other words, when parents or grandparents have poor health conditions, their children tend to inherit similar health challenges, indicating a cycle of intergenerational transmission of health disadvantage within families. The persistent and stubborn intergenerational transmission of health disadvantage among family members serves as a significant driver of health inequality [15]. Therefore, it becomes particularly important to seek effective means, strategies, and mechanisms to interrupt the transmission of health disadvantage.

Numerous studies have already investigated the intergenerational transmission of health between two generations [15–18], providing rich insights and deepening our understanding of health transmission within families. Health, being a multidimensional parameter, poses challenges in measurement as a singular indicator. Therefore, the researchers use different health measures to study the intergenerational transmission of health, such as self-rated health [16–19], birth weight [20–22], height, weight and BMIZ [13,17,18,23,24], life span [25–27], healthy behaviors [28–32]. In addition, some studies utilize multiple health indicators to study the intergenerational transmission of health [7,12,33,34], revealing a significant correlation between the health of parents and children.

However, focusing solely on the intergenerational transmission of health between two generations poses a significant limitation. This limitation lies in the inability to analyze the cross-generational transmission of health from grandparents to grandchildren, which may underestimate the persistence and severity of health disadvantage [22–37] and restrict a more comprehensive understanding of health transmission within families [15]. Mare emphasized the importance of investigating intergenerational transmission across three or more generations [35]. Since then, many scholars have explored intergenerational transmission across three generations in various aspects such as income, education, and social class [38–43], but research on the intergenerational transmission of health within three generations remains relatively scarce. Some studies have found that the weight or diabetes status of grandparents at birth can affect the birth weight and BMI of grandchildren [44–46], while the healthy behaviors of grandchildren are also closely related to the healthy behaviors of grandparents [47–49]. Moreover, grandparents’ eating disorder behavior influences grandchildren’ eating disorder behavior [50,51]. Recently, a study using Danish data discovered the intergenerational transmission of health and social disadvantage across three generations within Danish families, and it demonstrated that these disadvantage transmissions could be disrupted by increasing educational levels, highlighting the role of education in interrupting intergenerational disadvantage cycles [15].

Existing literature on the intergenerational transmission of health across three generations mainly focuses on developing countries [11,14]. As pointed out by [15], their evidence is limited to one country and one welfare system. Different countries have significant differences in terms of economic development, population structure, education quality, healthcare systems, and institutional cultures, which may lead to variations in the intergenerational transmission of health disadvantage and the role of education. Therefore, further research in China is necessary. China’s unique family culture and lifestyle [43,52,53] (such as intergenerational caregiving and three-generation living together, etc.) differ significantly from those from Western industrialized countries. These differences may influence the extent of intergenerational health transmission and remedial measures across three generations. Studying the intergenerational transmission of health and the disrupting role of education in Chinese families can provide empirical evidence for researching the impact of education on health in developing countries. By studying the intergenerational transmission of health disadvantage and the role of education, a deeper understanding of the mechanisms underlying the formation of health disadvantage can be attained. This understanding can provide factual evidence for the formulation and implementation of relevant policies.

Our study makes several significant contributions to the existing literature. Firstly, unlike existing research that primarily focuses on the intergenerational transmission of health between two generations, our study recognizes the importance of other members besides parents and highlights the persistence of health intergenerational transmission. Our study enriches the literature on health intergenerational transmission in developing countries, which has been relatively limited and predominantly focused on two generations. Secondly, while previous studies have mainly explored the correlation between education and intergenerational transmission of health disadvantage, our study takes a step further. We employ the Compulsory Education Law as an instrumental variable to assess the causal effect of education on the intergenerational transmission of health disadvantage. By employing this methodology, we contribute to the literature by offering a deeper understanding of the role of education in disrupting the transmission of health disadvantages across generations. Thirdly, there is a scarcity of literature that delves into the mechanisms through which education interrupts the intergenerational transmission of health disadvantage. Our study offers some mechanisms by exploring four key aspects: nutritional balance, healthy behaviors, social status, and income. By examining these mechanisms, we enhance the understanding of how education can disrupt the intergenerational transmission of health disadvantage. This analysis provides valuable insights that can inform the formulation and implementation of relevant policies aimed at interrupting the transmission of health disadvantage.

## Methodology

### Data

Our data is derived from the China Family Panel Studies (CFPS) [54], a survey conducted by the China Social Science Survey Center of Peking University. The survey covers 25 provinces, 105 counties and 16000 families. Since its inception in 2010, the CFPS has collected data over five rounds. The dataset comprises three types of questionnaires: village (neighborhood) questionnaire, household questionnaire, and individual questionnaire. For this study, we utilize the household questionnaire (economic questionnaire and family relationship questionnaire) and the individual questionnaire (adult questionnaire) from the CFPS waves of 2010, 2012, 2014, and 2016. These questionnaires detail information on various aspects such as household economy, family relationships, education level of each generation, health status, and income, making them suitable for studying intergenerational mobility.

To achieve our research goals, we conducted the following data processing steps: Firstly, using the adult code (referred to as G2, including mother and father both being alive), we linked the relevant information of their parents (referred to as G1, including grandmothers and grandfathers both being alive), and then use matched information of the two generations(G1 and G2) to match the pertinent information of their children (referred to as G3, including grandsons and granddaughters all being alive). In cases where there are multiple children within a family, each child was considered as a separate observation.) This approach allowed us to successfully establish two generations’ information. Secondly, we removed samples in which the age difference between any two generations is less than 15 years. This decision is based on the understanding that it is uncommon to have children before the age of 15. After applying these criteria, our final analytical sample consists in 5189 children with the corresponding parents and grandparents (5189+5189), for whom we gained comprehensive data on health, individual characteristics, and family background.

### Variables

#### Dependent and independent variables

Our study utilizes self-reported health status (SRHS) to measure the dependent variable (G3 health disadvantage), independent variable (G1 health disadvantage), and mediating variable (G2 health disadvantage). SRHS has been shown to be highly predictive of mortality even after controlling for other health measures, and outperforms other objective health measures [55–58]. However, as a robustness check, we supplement our analysis by constructing a mental health index. The self-reported health status variable is obtained from the answers provided by G1\G2\G3 to the following questions: "How do you consider your physical health"? The response scale ranges from 1 to 5, with response categories as follows: 1 = very good, 2 = good, 3 = fair, 4 = poor, and 5 = very poor. Considering the varying health levels within each generation, we measure the health variable of G1\G2\G3 using SRHS-for-age and gender z scores (same gender, age of each generation). The z scores for G1\G2\G3 are calculated as the deviation of the individual’s observed SRHS from the mean value of the reference population, divided by the standard deviation of the group in the following way: z score = (observed value−mean reference value) \standard deviation of a reference population. We then operationally defined a health disadvantage as 25% of z score who accounted for the unhealthiest, denoted as 1, while others are denoted as 0.

#### Covariates

We include several individual and family background characteristics as control variables in our analysis. Specifically, we control for G2 Completing compulsory education (1 = yes, 0 = no). Additionally, we also control for gender (1 = male, 0 = female), age, age squared (Adding the age squared allows article to model the effect differing ages, rather than assuming the effect is linear for all ages.) and hukou type (1 = Agricultural Hukou, 0 = Non-agricultural Hukou, Chinese citizens are labelled as having an agricultural or non-agricultural household registration. It is the system of household registration used in mainland China.) for G1, G2, and G3. In addition, we control for marital status (1 = Married, 0 = Other) for G1 and G2 (Because the average age of G3 is not legally old enough to marry. Therefore, this article does not control the marital status of G3). Other control variables include G2 number of children and household net income per capita.

#### Descriptive statistics

We use STATA.17 for statistical analysis. Table 1 presents the variable distribution within our analytical sample, which consists of 5189 G1-G2-G3 triads with valid data for all measurement. The G1 generation has an average self-reported health status of 3.513 (between fair and poor), with 40.5% of them being male. 81.1% of them come from agricultural hukou families, and 60.2% of them are married. The average age of this generation is 72.38 years old. The G2 generation has an average self-reported health status of 2.701 (between good and fair). Around 80.3% of them are from agricultural hukou families, with 89.9% of them being male (CFPS primarily collects information from core family members who live together and have an economic relationship, and core families are mainly male-dominated in China. Therefore, the average male proportion is relatively high). Additionally, 93.6% of the G2 are married. The average age of this generation is 45.45 years old, and they have an average of about two children. The G2 generation also holds an average of 7.192 years of schooling, with 55.8% of them having completed compulsory education. Lastly, the G3 generation reports an average self-rated health status of 2.109 (good). Within this generation, 78.4% of them come from agricultural hukou families, 55.4% of them are male, and the average age is 20.74 years old.

**Table 1:** Descriptive statistics.

	Variable	Mean	Proportion	SD	Min	Max
Grandparents (G1)	G1 Self-reported health status	3.513		1.272	1	5
	G1 Health disadvantage (Yes = 1)		24.9%			
	G1 Age	72.38		6.899	56	93
	G1 Gender (Male = 1)		40.5%			
	G1 Hukou (Agricultural hukou = 1, other = 0)		81.1%			
	G1 Marital status (Married = 1, other = 0)		60.2%			
Parents (G2)	G2 Self-reported health status	2.701		1.212	1	5
	G2 Health disadvantage (Yes = 1)		25.0%			
	G2 Compulsory education (Yes = 1)		55.8%			
	G2 Years of schooling(years)	7.192		3.957	0	19
	G2 Age	45.45		4.771	35	63
	G2 Gender (Male = 1)		89.9%			
	G2 Hukou (Agricultural hukou = 1, other = 0)		80.3%			
	G2 Marital status (Married = 1, other = 0)		93.6%			
	Number of G2 children	2.095		0.899	1	7
Grandchildren (G3)	G3 Self-reported health status	2.109		0.966	1	5
	G3 Health disadvantage (Yes = 1)		24.9%			
	G3 Age	20.74		3.952	16	35
	G3 Gender (Male = 1)		55.4%			
	G3 Hukou (Agricultural Hukou = 1, other = 0)		78.4%			
Household net income per capita	The logarithm of household net income per capita	8.801		1.172	0.288	12.073

### Analytical strategy

**Fig 1:**
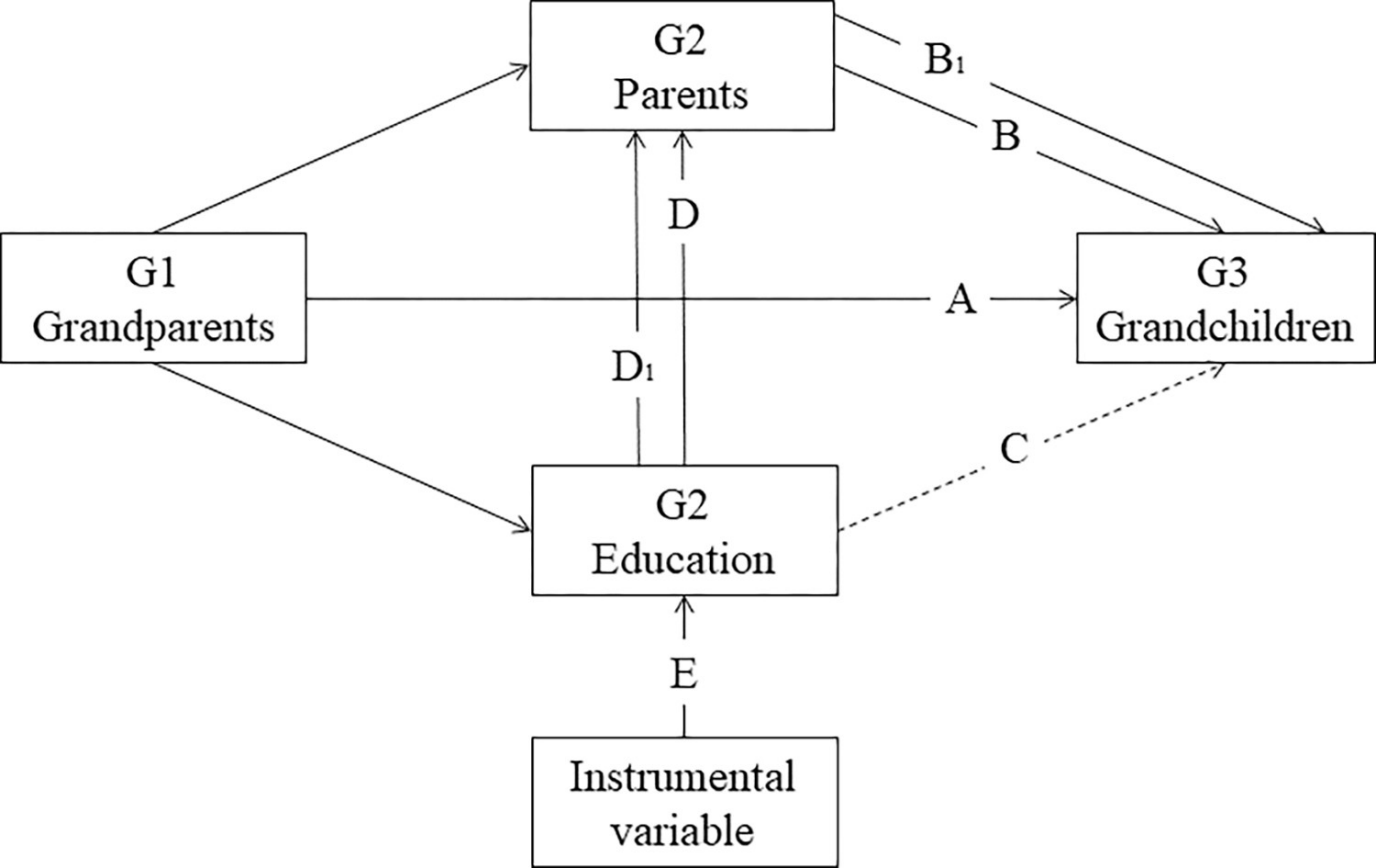
Education disrupts the intergenerational transmission of health disadvantage across three generations.

In this study, we employ a Logit model to investigate the intergenerational transmission of health disadvantage. Our analysis includes a three-generation model to examine two specific associations. Firstly, we examine the direct relationship between the health disadvantage of G1 and the subsequent health disadvantage of G3 without considering the influence of G2 health disadvantage and other relevant characteristics (G1→G3, shown as A in Fig 1). Secondly, we assess the indirect association between the health advantage of G1 and the health disadvantage of G3 while controlling for G2 health disadvantage and relevant characteristics (G1→G2→G3, depicted as B in Fig 1).

To examine the role of G2 completing compulsory education in mediating the association between G1 health disadvantage and G3 health advantage, Fig 1 indicates the position and role of education in the health transmission chain. It disrupts the intergenerational transmission of disadvantage from G1 to G2 (arrow path D), indirectly disrupts the intergenerational transmission of health disadvantage from G2 to G3 by improving G2 health, as well as disrupts the cross-generational disadvantage transmission from G1 to G3 (arrow paths C and DB, but this article examines DB path, path C is represented by a dotted line). We refer to this weakening or disrupting effect of education within the transmission chain as the "disrupting effect of education". To assess the magnitude of educational disruption in various transmission relationships, we adopt the approach of the KHB [59,60] method to decompose the contributions of education to the transmission.

It is important to note that the disrupting effect of education may be influenced by factors such as the family environment, unobservable missing variables, and other potential residual confounding. However, when it comes to policy-making, it is necessary to assume that education has a causal effect on health [15]. To establish a more convincing the causal effect of education on the intergenerational transmission of health disadvantage, we further use the Compulsory Education Law as an instrumental variable (IV) to examine whether G2 completing compulsory education can indeed help disrupt the cross-intergenerational transmission of health disadvantage (arrow path D_1_B_1_).

The Law on Nine-Year Compulsory Education was implemented on July 1st, 1986, with the ultimate goal of establishing a nine-year cycle of mandatory education. This law explicitly outlines the following provisions: Firstly, children who have reached the age of six must enroll in and receive compulsory education for the designated period. Secondly, the duration of compulsory education spans nine years, covering primary and junior high school. Thirdly, the specific implementation time is determined by individual provinces based on local conditions. As a result, individuals older than 15 years old are not subject to the Compulsory Education Law. With this information, we can determine the birth month and year of the earliest cohort impacted by the implementation of the Compulsory Education Law. The group affected by the law is denoted as 1, while others are denoted as 0.

Additionally, we investigate a discussion on potential mechanisms by which education interrupts the transmission of health disadvantage, and examine these mechanisms in detail.

### Empirical model

A large amount of literature defines intergenerational transmission in terms of intergenerational association [15,61]. In our research, we employ a Logit model to examine whether there is a significant direct or indirect association between health disadvantage of G1 and G3, both with and without controlling for G2 health disadvantage and relevant characteristics. Additionally, we aim to determine whether G2’s completion of compulsory education can disrupt the health disadvantage association between G1 and G3. The model specifications are as follows:

$$Y_{i}=\alpha_{0}+\alpha_{1}D_{i}+\alpha_{2}C_{i}+R+A+T+\mu_{i}$$ (1)

$$Y_{i}=\beta_{0}+\beta_{1}D_{i}+\beta_{2}C_{i}+R+A+T+\mu_{i}$$ (2)

$$Y_{i}=\alpha_{0e}+\alpha_{1e}D_{i}+{\alpha_{3e}E}_{i}+\alpha_{2e}C_{i}+R+A+T+\mu_{i}$$ (3)

Model (1) to examine the direct relationship between the health disadvantage of G1 and the health disadvantage of G3 (G1→G3, shown as A in Fig 1): Where Y_i_ is the measure of health disadvantage for G3; D_i_ is the measure of G1 health disadvantage; C_i_ indicates a series of control variables measured separately for G1(including age, age square, gender, hukou type, marital status and G3 (including age, age square, gender, hukou type); R indicates the regional fixed effects (divided into four regions: East, Central, West, and Northeast based on G2’s province). It mainly aims to control for the effects of regional-specific diseases and macro-environmental factors. A indicates a series of age cohort effects for G1 and G3 (Age cohort effect refers to the influence that peer groups have on the development of group members by living in similar socio-cultural environments and experiencing similar historical events. Age cohort is grouped into ten-year periods for each generation), which control peers who have common factors affecting education level; T indicates the time-fixed effect (We use waves of 2010, 2012, 2014, and 2016, being to mixed panel data. Time fixed effects allow controlling for underlying observable and unobservable systematic differences between observed time units), which controls the problem of missing variables that do not vary with individuals but change over time; U_i_ indicates error term.

Model (2) to assess the indirect association between the health advantage of G1 and the health disadvantage of G3 while controlling for G2 health disadvantage and relevant characteristics (G1→G2→G3, depicted as B in Fig 1): We add a series of control variables for G2, including G2 health disadvantage, age, age square, gender, hukou type, number of G2 children, marital status. All other control variables are as same as Model (1).

Model (3) to examine whether G2 completing compulsory education can disrupt the cross-generational disadvantage transmission from G1 to G3 (depicted as DB in Fig 1): We add G2 compulsory education to Model (2), *E*_*i*_ in Model (3) indicates whether G2 completing compulsory education or not. When *α*_3*e*_ is negative and the coefficient of *α*_1*e*_ is smaller than that of *α*_1_, indicating that G2 completing compulsory education interrupts the intergenerational transmission of health disadvantage. All other Controlled variables are as same as Model (2).

The above analysis which examines the role of education in the transmission chain is based on regression methods. However, there are potential issues with confounding variables and omitted variables. For example, the family environment and certain unobservable long-term patterns may simultaneously influence both the transmission of health disadvantage within families and the education of intermediate generations. This can lead to a false perception of the impact of education observed in correlational regressions. In order to address potential endogeneity concerns in the analysis, this study uses the Compulsory Education Law as an instrumental variable (IV) for G2 completing compulsory education to assess causal effect of education on the intergenerational transmission of health disadvantage. Instrumental variable (IV) method plays an important role in the study of causality, especially in the face of endogenous problems [62,63]. The model specifications are as follows:

$$E_{i}=\gamma_{1}D_{i}+\gamma_{3}IV+\gamma_{2}C_{i}+A+T+\eta_{i}$$ (4)

$$Y_{i}=\alpha_{0e}+\alpha_{1e}D_{i}+\alpha_{3e}E_{i}+\alpha_{2e}C_{i}+R+A+T+\mu_{i}$$ (5)

Model (4) is the first stage of instrumental variable (IV) (shown as E in Fig 1). E_i_ indicates whether G2 have completed nine years of compulsory education. D_i_ is the measure of G1 health disadvantage. IV is instrumental variable referring to Compulsory Education Law. All other control variables are as same as Model (2).

Model (5) is the second stage of instrumental variable (IV) (shown as D_1_B_1_ in Fig 1). D_i_ is the measure of G3 health disadvantage. E_i_ indicates G2 whether have completed nine years of compulsory education. All other control variables are as same as Model (4).

The goodness-of-fit of the regressions assessed trough the R^2^ coefficients have been adjusted in all Models.

### Result

Table 2 presents the estimated results of intergenerational health disadvantage transmission, with the reported coefficients representing the marginal effects. Column (1) presents the estimate of the association between G1 health disadvantage and G3 health disadvantage without considering G2 characteristic variables. The estimated coefficient for intergenerational health disadvantage for G1 is 0.397 (p < 0.01), indicating that G1 health disadvantage significantly increases the probability of G3 health disadvantage by 3.97 percentage points. The result of Column (1) supports path A (G1→G3) in Fig 1.

**Table 2:** Education interrupts the intergenerational transmission of health disadvantage.

	(1)	(2)	(3)	(4)	(5)	(6)
Dependent variable	G3 Health disadvantage	G3 Health disadvantage	G3 Health disadvantage	G3 Health disadvantage	Instrumental variable	G3 Health disadvantage
Transmission	G1→ G3	G1→G2→G3	G1→G2→G3	G1→G2→G3	First stage	2SLS
Independent variable						
G1Health disadvantage	0.0397***	0.0349***	0.0330**	0.0328**	-0.0025	0.1097**
	(0.0136)	(0.0135)	(0.0135)	(0.0136)	0.0153	(0.0471)
Mediating variables						
G2 Health disadvantage		0.0834***	0.0860***	0.0833***	-0.0928***	0.1937***
		(0.0131)	(0.0131)	(0.0131)	(0.0152)	(0.0706)
G2 Completing compulsory education				-0.0274**		-1.0428*
				(0.0126)		(0.5864)
Instrumental variable					0.1366***	
					(0.0258)	
Covariates						
G1 Age	0.0226	0.0183	0.0113	0.0115	0.0156	0.0410
	(0.0241)	(0.0240)	(0.0245)	(0.0245)	0.0276	(0.0864)
G1 Age^2	-0.0001	-0.0001	-0.0001	-0.0001	-0.0001	-0.0002
	(0.0002)	(0.0002)	(0.0002)	(0.0002)	0.0002	(0.0006)
G1 Gender	0.0127	0.0135	0.0155	0.0150	-0.0128	0.0311
	(0.0127)	(0.0127)	(0.0128)	(0.0128)	0.0141	(0.0445)
G1 Hukou	0.0083	0.0055	0.0178	0.0164	-0.0535**	-0.0058
	(0.0210)	(0.0208)	(0.0243)	(0.0243)	0.0256	(0.0847)
G1 Marital status	-0.0023	0.0011	-0.0001	0.0025	0.0964***	0.1083
	(0.0138)	(0.0138)	(0.0139)	(0.0139)	(0.0151)	(0.0744)
G2 Age			0.0005	0.0043	0.1812***	0.1508
			(0.0303)	(0.0304)	0.0338	(0.1307)
G2 Age^2			-0.0000	-0.0001	-0.0017***	-0.0015
			(0.0003)	(0.0003)	0.0004	(0.0013)
G2 Gender			0.0203	0.0222	0 .0569**	0.1388*
			(0.0209)	(0.0208)	0.0227	(0.0790)
G2 Hukou			-0.0403	-0.0428	-0.0909***	-0.2245**
			(0.0266)	(0.0266)	0.0297	(0.1045)
G2 Marital status			-0.0308	-0.0290	0.0707**	-0.0056
			(0.0243)	(0.0242)	0.0273	(0.0947)
Number of G2 children			0.0125	0.0110	-0.0592***	-0.0196
			(0.0076)	(0.0077)	0.0084	(0.0439)
G3 Age	-0.0871***	-0.0913***	-0.1012***	-0.1024***	-0.0324	-0.3612***
	(0.0205)	(0.0205)	(0.0217)	(0.0217)	0.0246	(0.0790)
G3 Age^2	0.0019***	0.0020***	0.0023***	0.0023***	0.0004	0.0077***
	(0.0004)	(0.0004)	(0.0005)	(0.0005)	0.0005	(0.0017)
G3 Gender	0.0091	0.0082	0.0124	0.0117	-0.0202	0.0186
	(0.0120)	(0.0120)	(0.0122)	(0.0122)	0.0135	(0.0441)
G3 Hukou	0.0060	0.0097	0.0216	0.0192	-0.0717***	0.0062
	(0.0202)	(0.0201)	(0.0237)	(0.0237)	0.0264	(0.0955)
The logarithm of household net income per capita	0.0056	0.0064	0.0073	0.0083	0.0350***	0.0587**
	(0.0056)	(0.0056)	(0.0057)	(0.0057)	(0.0061	(0.0281)
F					23.62	
Age cohort effect	Yes	Yes	Yes	Yes	Yes	Yes
Regional fixed effect	Yes	Yes	Yes	Yes	Yes	Yes
Time-fixed effect	Yes	Yes	Yes	Yes	Yes	Yes
N	5189	5189	5189	5189	5072	5072
Adj. R^2^	0.0187	0.0255	0.0271	0.0279	0.1304	

All regressions are estimated by Logit. Each term between brackets () corresponds to the standardized coefficients. Cluster-corrected standard errors in parentheses

*** p<0.01

** p<0.05

*p<0.1.

Columns (2) incorporates G2 health disadvantage as a mediating variable to estimate the intergenerational transmission of G1→G2→G3. In columns (3), the results from these two columns reveal that G1 health disadvantage significantly increases the probability of G3 health disadvantage, with indirect effects of approximately 3.49 and 3.3 percentage points (p<0.01), which supports path B(G1→G2→G3) in Fig 1. Additionally, G2 health disadvantage also significantly increases the probability of G3 health disadvantage by approximately 8.34 and 8.6 percentage points. Specifically, the estimated coefficient for G1 health disadvantage in columns (2) is slightly smaller than that in columns (1), suggesting that the mediating variable of G2 health disadvantage absorbs some of the effects of the transmission from G1 to G3.

In Column (4) shows that G1 health disadvantage increases significantly the probability of G3 health disadvantage by 3.28 percentage points, while G2 health disadvantage increases significantly the probability by 8.33 percentage points. Remarkably, the results from Column (4) indicate that G2 completing compulsory education can reduce the probability of G3 experiencing health disadvantage by 2.74 percentage points (supports path DB in Fig 1).

**Fig 2:**
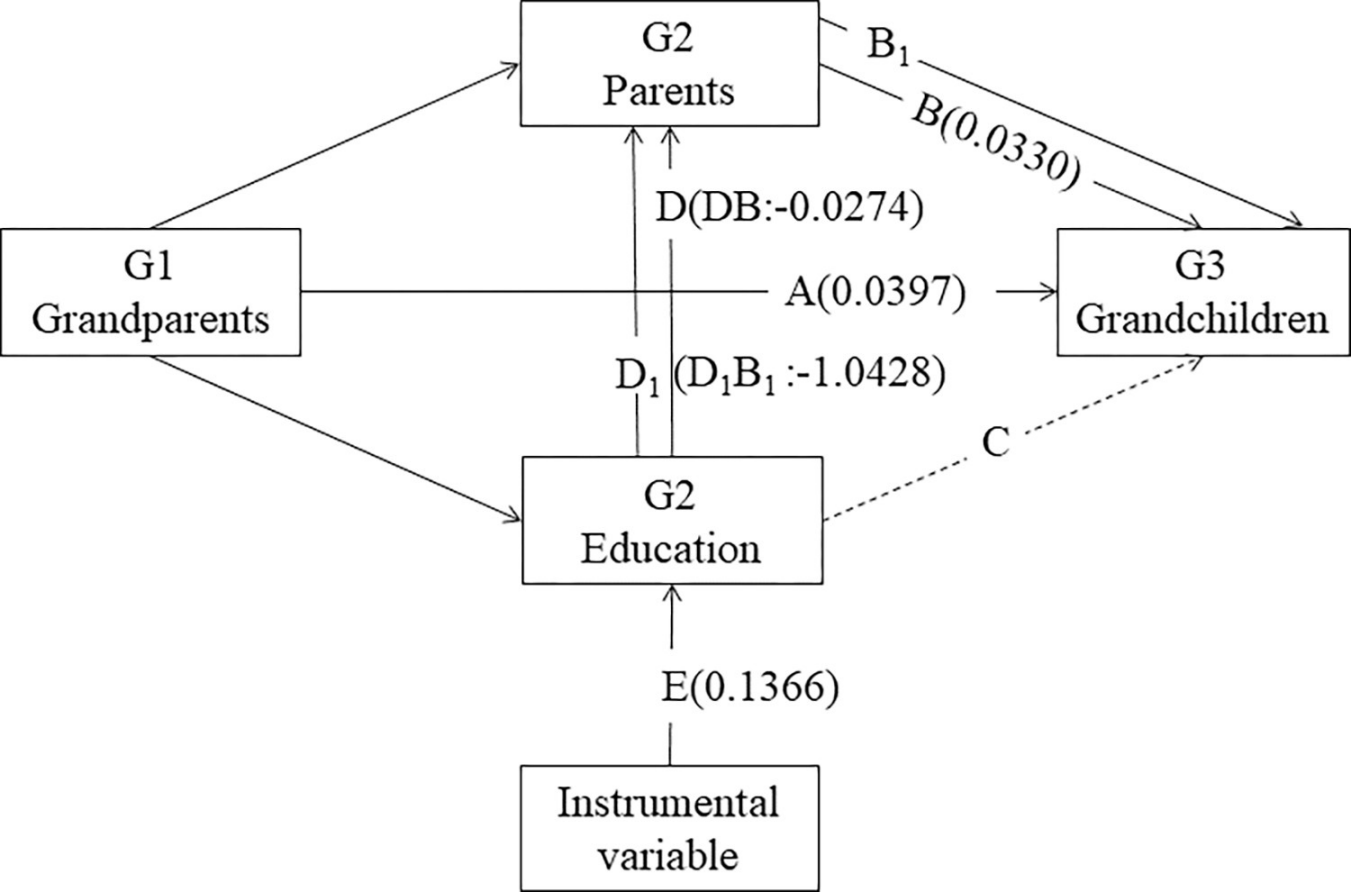
Education disrupts the intergenerational transmission of health disadvantage across three generations with the coefficient of each path.

Column (5) in Table 2 shows that the instrumental variable F-statistic is 23.62, which is exceeding the threshold of 10. This indicates the absence of a weak instrumental variable problem. The endogeneity test (Wald, P = 0.0753) rejects the null hypothesis of no endogeneity at a 10% significance level. Thus, instrumental variables pass the endogeneity and weak instrument tests. The regression results from the first stage are presented in Table 2 (Column 5), indicating a significant positive impact of the implementation of the compulsory education law on G2 completing compulsory education (p<0.01). The second stage regression results show (Column 6) the coefficient for G2 completing compulsory education increases significantly when instrumental variable is used in the regression analysis. Considering the endogeneity of G2 completing compulsory education, it is found that completing nine years of compulsory education significantly reduces the probability of both themselves and the next generation experiencing the health disadvantage. This implies that G2 completing nine years of compulsory education indeed interrupts the intergenerational transmission of health disadvantage cross three generations. Path plot with the parameter estimates shows in Fig 2.

### Robustness test

Next, we proceed to examine the robustness of the intergenerational transmission of health disadvantage by altering the measurements of key variables, employing different estimation methods, and utilizing alternative health indicators. Table 3 reports the robustness estimation results for the intergenerational transmission of health disadvantage across these four scenarios, with the reported coefficients indicating marginal effects.

**Table 3:** Robustness test.

	(1)	(2)	(3)	(4)
Dependent variable	G3 Health disadvantage	G3 Health disadvantage	G3 Health disadvantage	G3 Health disadvantage
Transmission	G1→ G3	G1→G2→G3	G1→G2→G3	G1→G2→G3
*Panel A*: 20% of z score				
Independent variable: G1 Health disadvantage	0.0348***	0.0302**	0.0294**	0.0291**
	(0.0133)	(0.0132)	(0.0133)	(0.0133)
Mediating variable: G2 Health disadvantage		0.0757***	0.0766***	0.0742***
		(0.0127)	(0.0127)	(0.0128)
Mediating variables: G2 Completing Compulsory education				-0.0202*
				(0.0115)
*Panel B*: Defines individuals with "poor" and "very poor" as the health disadvantage group				
Independent variable: G1 Health disadvantage	0.0289***	0.0247***	0.0241***	0.0240***
	(0.0074)	(0.0074)	(0.0074)	(0.0074)
Mediating variable: G2 Health disadvantage		0.0387***	0.0407***	0.0393***
		(0.0074)	(0.0074)	(0.0074)
Mediating variable: G2 Completing compulsory education				-0.0151**
				(0.0073)
*Panel C*: OLS estimation (self-reported health five categories)				
Independent variable: G1 Health disadvantage	0.0713***	0.0556***	0.0542***	0.0543***
	(0.0107)	(0.0107)	(0.0108)	(0.0108)
Mediating variable: G2 Health disadvantage		0.1107***	0.1131***	0.1118***
		(0.0114)	(0.0115)	(0.0116)
Mediating variable: G2 Completing compulsory education				-0.0519**
				(0.0263)
N	5189	5189	5189	5189
*Panel D*: Mental health indicators (depression)				
Independent variable: G1 Mental health disadvantage	0.0687***	0.0465**	0.0509**	0.0480**
	(0.0196)	(0.0203)	(0.0203)	(0.0203)
Mediating variable: G2 Mental health disadvantage		0.0940***	0.0945***	0.0905***
		(0.0198)	(0.0198)	(0.0199)
Mediating variable: G2 Completing compulsory education				-0.0427**
				(0.0187)
N	2333	2254	2254	2254

All regressions are estimated by Logit. All Models controlling for variables are as same as Table 2. Each term between brackets () corresponds to the standardized coefficients. Cluster-corrected standard errors in parentheses

*** p<0.01

** p<0.05

*p<0.1.

Panel A in Table 3 presents the regression results using a new operational definition, where a health disadvantage is defined as 20% of individuals with the highest z score for unhealthiness. We continue to use Logit estimation for this analysis. Panel A shows coefficients for the two key variables (G1 Health disadvantage and G2 Health disadvantage) are significantly positive (p<0.01), while G2 completing compulsory education decreases the probability of G3 experiencing health disadvantage by 2.02 percentage points.

In Panel B, we classify individuals with "poor" and "very poor" health status as the health disadvantage group. The results of Panel B reveal that G1 or G2 health disadvantage significantly increases the probability of G3 health disadvantage, while G2 completing compulsory education increases the probability of the health of G3 being to "very good" and decreases the probability of being to "good/average/bad/very bad".

In Panel C, we estimate self-reported health by categorizing responses into five categories: "very poor/poor/fair/good/very good", using OLS estimation. The results in Panel C show that G1 health disadvantage significantly increases the probability of G3 health disadvantage, while G2 health disadvantage also significantly increases the probability of G3 health disadvantage and G2 completing compulsory education leads to better health for G3

Finally, Panel D uses mental health indicators (depression) as an alternative measure of health. We then operationally defined a health disadvantage as 25% of individuals with the highest level of mental unhealthiness. The estimated coefficient for the two key variables (G1 health disadvantage and G2 health disadvantage) are both significant. G2 health disadvantage also significantly increases the probability of G3 health disadvantage, while G2 Completing compulsory education reduces the probability of G3 experiencing health disadvantage. These results are completely consistent with the baseline regression presented in Table 2.

### Heterogeneity analysis

Table 4 presents the results of heterogeneity analysis based on G3 Hukou type, gender, and household net income per capita. We divide full sample into two subsamples: one with Agricultural Hukou and the other with Non-Agricultural Hukou in Column (1) and (2). In Columns (3) and (4), we divide full sample into two subsamples based on high income and low income by household net income per capita. Finally, Columns (5) and (6) divide full sample into two subsamples based on grandsons and granddaughter according to G3’s gender. The findings reveal that G1 health disadvantage increases the probability of G3 health disadvantage by 4.83 percentage points, but only when G3 comes from an Agricultural Hukou. Additionally, as shown in Column (1), completing compulsory education for G2 can significantly decrease intergenerational transmission of health disadvantage, but only for G3 coming from Agricultural Hukou. Furthermore, Columns (3) and (4) in Table 4 reveal that only the low-income families subsample exhibits a positive and statistically significant coefficient between G1 health disadvantage and G3 health disadvantage. Completing compulsory education for G2 can significantly decreases the association in low-income families. Columns (5) and Column (6) show there are positive association between G1 health disadvantage and grandsons’ health disadvantage only, while completing compulsory education for G2 significantly reduces this association.

**Table 4:** Heterogeneity analysis.

	(1)	(2)	(3)	(4)	(5)	(6)
Dependent variable	Heterogeneity by G3 hukou		Heterogeneity by G3 Family income		Heterogeneity by G3 gender	
	Agriculture	Non-Agriculture	lower	Higher	Grandsons	Granddaughters
Independent variable						
G1 Health disadvantage	0.0483***	-0.0382	0.0573***	-0.1005	0.0552***	-0.0044
	(0.0151)	(0.0322)	(0.0161)	(0.1416)	(0.0182)	(0.0207)
Mediating variables						
G2 Health disadvantage	0.0800***	0.0814***	0.0878***	0.4277***	0.0835***	0.0804***
	(0.0149)	(0.0278)	(0.0158)	(0.1316)	(0.0180)	(0.0196)
G2 Completing compulsory education	-0.0305**	-0.0200	-0.0260*	-0.1269	-0.0301*	-0.0260
	(0.0139)	(0.0321)	(0.0151)	(0.1255)	(0.0174)	(0.0184)
N	4066	1123	3375	1814	2824	2365
Adj.R^2^	0.0320	0.0466	0.0299	0.0453	0.0346	0.0416

All regressions are estimated by Logit. All Models controlling for variables are as same as Table 2; Each term between brackets () corresponds to the standardized coefficients. Cluster-corrected standard errors in parentheses

*** p<0.01

** p<0.05

*p<0.1.

### Education interrupts the intergenerational transmission of health disadvantage: KHB decomposition

Table 5 illustrates the extent to which G2’s health disadvantage explains the intergenerational transmission of health disadvantage from G1 to G3, and assess the contribution of G2 education in disrupting the grandparents’ effects. Table 5 shows that the total effect, direct effect, and indirect effect are all statistically significant at conventional levels, suggesting that both G2 health disadvantage and education significantly mediate the association of the health disadvantage between G1 and G3. It can be observed that G2 education accounts for a similar percentage (1%) in explaining the intergenerational health transmission effects. 12%-25% of the total effect can be attributed to the combination of G2 education and health disadvantage.

**Table 5:** KHB decomposition.

	(1)	(2)	(3)	(4)	(5)
Dependent variable	G3 Health disadvantage	G3 Health disadvantage	G3 Health disadvantage	G3 Health disadvantage	G3 Health mental disadvantage
Total effect	0.0383***	0.0332**	0.0279***	0.0703***	0.0471**
	(0.0135)	(0.0133)	(0.0073)	(0.0106)	(0.0200)
Direct effect	0.0334**	0.0289**	0.0239***	0.0543***	0.0347*
	(0.0135)	(0.0133)	(0.0074)	(0.0108)	(0.0204)
Indirect effect	0.0049***	0.0043***	0.0040***	0.0160***	0.0124***
	(0.0000)	(0.0000)	(0.0000)	(0.0000)	(0.0000)
Corresponding percentage (G2 education and health)	12.90%	12.96%	14.32%	22.70%	26.29%
Corresponding percentage (G2 education)	0.52%	0.77%	0.35%	0.15%	1.34%
Corresponding percentage (G2 health disadvantage)	12.38%	12.19%	13.98%	22.55%	24.95%
Control variables	Table 2 (4)	Table 3 *Panel A* (4)	Table 3 *Panel B* (4)	Table 3 *Panel C* (4)	Table 3 *Panel D* (4)
N	5189	5189	5189	5189	2254
Adj.R^2^	0.03	0.03	0.07	0.17	0.05

All regressions are estimated by Logit besides Column (4), which uses OLS regression; All Models controlling for variables are as same as Table 2; Each term between brackets () corresponds to the standardized coefficients; Columns (1) correspond to columns (4) in Table 2, while columns (2) to (5) correspond to Panel A (4) to Panel D (4) in Table 3; Cluster-corrected standard errors in parentheses

*** p<0.01

** p<0.05

*p<0.1.

## Mechanism analysis

Due to data limitation, our study focuses on how G2 completing compulsory education influences their own and next-generation’s health through G2 socioeconomic status (income and social status), healthy behaviors, and nutritional balance. The coefficient of Column (1) in Table 6 (Panel A) indicates that G2 completing compulsory education significantly increases their own nutritional balance by 22.39 percentage points. Moreover, the coefficient of Column (2) (Panel A) suggests that G2 nutritional balance significantly decrease their own health disadvantage probability by 2.1 percentage points. Similarly, in Column (3) (Panel A), it indicates a decrease of 1.33 percentage points in health disadvantage probability for the next generation (G3). Similarly, the other mechanisms can be interpreted in a similar way. G2 completing compulsory education significantly increases own nutritional balance, reduces their negative healthy behaviors (Have you ever smoked), raises their income levels (measured by annual income), and improves the probability of higher social status. On the contrary, negative healthy behaviors increase the probability of health disadvantage for both G2 and G3. Additionally, G2’s nutritional balance, higher income and high social status decrease significantly the probability of health disadvantage for themselves and G3. This evidence supports the notion that G2 completing compulsory education improves individuals’ nutritional balance, health behaviors, income, and social statue, thus enhancing the health of themselves and G3.

**Table 6:** Mechanism test.

	(1)	(2)	(3)	(4)	(5)	(6)
Panel A: Nutritional balance and healthy behaviors						
Dependent variables	G2 Nutritional balance	G2 Health disadvantage	G3 Health disadvantage	G2 Have smoked	G2 Health disadvantage	G3 Health disadvantage
G2 Complete Compulsory education	0.2339***			-0.0859**		
	(0.0563)			(0.0374)		
G2 Nutritional balance		-0.0210***				
		(0.0040)				
G3 Nutritional balance			-0.0133**			
			(0.0065)			
G2 Have smoked					0.1642***	0.1042**
					(0.0542)	(0.0489)
G2 Health disadvantage			0.0740***			0.0350
			(0.0194)			(0.0438)
N	2548	2838	2518	438	414	411
Adj.R^2^	0.1470	0.0156	0.0449	0.2033	0.0511	0.0846
Panel B: Income and social status						
Dependent variables	G2 High income	G2 Health disadvantage	G3 Health disadvantage	G2 High Social status	G2 Health disadvantage	G3 Health disadvantage
G2 Complete compulsory education	0.0323**			0.0279**		
	(0.0140)			(0.0138)		
G2 High income		-0.0810***	-0.0365*			
		(0.0222)	(0.0216)			
G2 High social status					-0.1169***	-0.0474***
					(0.0125)	(0.0131)
G2 Health disadvantage			0.0930***			0.0763***
			(0.0172)			(0.0137)
N	3107	3089	3089	4963	4884	4859
Adj.R^2^	0.2063	0.0101	0.0265	0.0277	0.0210	0.0260

All regressions are estimated by Logit besides Column (1)in Panel A which uses OLS regression; Column (1), Column (2),Column (3) and Column (4) Controlling for variables: age, age square, gender, marital status, time-fixed effect, Regional fixed effect and age cohort effect of G2;Column (3) and Column (6) Controlling for variables: age, age square, gender, marital status, time-fixed effect, Regional fixed effect and age cohort effect of G2 and G3; Each term between brackets () corresponds to the standardized coefficients; Cluster-corrected standard errors in parentheses

*** p<0.01

** p<0.05

*p<0.1.

## Discussion and conclusion

Health transmission not only occurs between two generations but also across multiple generations. Therefore, it is necessary to expand the research on family health transmission from two generations to three or even more generations. This study uses data from the China Family Panel Studies (CFPS) to examine the transmission of health disadvantage across three generations. The results indicate significant intergenerational transmission of health disadvantage within Chinese families, including both direct and indirect transmission from G1 to G3. Specifically, the findings demonstrate that G1 health disadvantage contributes to an increase of 3.97 percentage points for probability of health disadvantage for G3. After controlling for parental health disadvantage and relevant variables, the intergenerational transmission analysis shows that G1 health disadvantage contributes to an increase of approximately 3.3 percentage points in the health disadvantage of G3. One possible explanation for these results is that intergenerational caregiving and cohabitation with G1 can lead to greater direct and indirect health transmission to G3. We find G2 health disadvantage plays an important mediating role in the relationship between G1 and G3 health disadvantage, helping to explain the continuity in health disadvantage across generations. These results indicate that improving G2 health also disrupts the health disadvantage between G1 and G3. These findings emphasize the necessity of investigating the transmission of health disadvantage across three or more generations. It is evident that solely focusing on the intergenerational transmission between two generations would significantly underestimate the severity of health disadvantage transmission. Therefore, to obtain a comprehensive understanding of health disparities and their transmission, it is necessary to investigate the transmission of health disadvantage across three or more generations.

Existing literature on industrialized Western countries suggests that education plays a significant role in disrupting the transmission of health disadvantage, but this evidence is primarily based on correlation analysis in developed countries. However, the evidence from China only partially supports this conclusion. Our findings indicate that when G2 completes compulsory education, it can indeed disrupt the transmission of health disadvantage from G1 to G3. We use the Compulsory Education Law as an instrumental variable to measure G2’s completion of compulsory education and further confirm that education can disrupt the intergenerational transmission of health disadvantage. However, it is important to note that the magnitude of this disrupting effect is relatively limited, accounting for only about 1% and much lower compared to what is observed in industrialized Western countries. One possible explanation is that the education level examined in our study refers to G2 completing nine years of compulsory education, which is a widely but relatively low level of education. Nevertheless, this should not undermine the importance of improving education. Our study provides evidence that the completion of compulsory education by G2, as a crucial determinant of health, significantly reduces the probability of health disadvantage for both themselves and their children. Although the disrupting effect of education is limited, these findings suggest that relying solely on improving education is insufficient, and additional approaches beyond education are needed to break the chain of disadvantage transmission. For example, improving the health of the middle generation, equitable healthcare and welfare, and enhancing housing conditions may prove to be more effective in preventing the health disadvantage transmission.

Our heterogeneity analysis reveals that parents completing compulsory can significantly disrupts intergenerational transmission of health disadvantage only grandchildren coming from Agricultural Hukou or low-income family. The explanations for these results above are as follows: Firstly, the accessibility of medical resources plays a role. For Agricultural Hukou residents, the disease rate of G3 in low-income families is significantly higher than that in high-income families [64], and the constraints of medical infrastructure make Agricultural Hukou residents more prone to intergenerational transmission of health disadvantage. Secondly, due to Agricultural Hukou residents’ income is low, low-income families constrained by their financial situation, invest less in the health of their children. Thirdly, the relatively low educational level of Agricultural Hukou residents’ parents may result in a neglect of health education for their offspring to some extent. This also indirectly indicates the correlation between the degree of intergenerational health transmission and income disparity. Therefore, improving education, medical and health conditions are effective means to disrupt intergenerational transmission of health disadvantage in low-income and Agricultural Hukou families. Additionally, there are son preferences on disrupting effect of health disadvantage three-generational transmission in China. One explanation is that son preference is deeply embedded in Chinese social and cultural values [65], resulting in grandparents passing down their health primarily to grandsons.

The impact of education on individual and intergenerational health, as well as its ability to disrupt transmission, may vary due to different environments. However, there are several mechanisms and pathways through which education can influence health: Firstly, higher educational attainment is likely to increase individual’s health knowledge, enabling individuals to adopt healthier behaviors and make better health decisions. Secondly, education enhances individual’s health consciousness and attitudes, leading individuals to pay more attention to their own and their family members’ health. This fosters the development of healthy behaviors and lifestyles and positively influences family members. Thirdly, education strengthens cognitive abilities and development, equipping individuals with better problem-solving skills and the ability to cope with stress and adverse life events. This can have a positive impact on the health of individuals and their families. Fourth, education improves individual’s socioeconomic status by facilitating access to better occupations and work, social networks, higher income, and social status. This enables individuals to have the capacity to invest more in their own health or obtain better healthcare services for themselves and their families. Fifth, individuals with higher education levels tend to have access to higher-quality partners, focus on scientific prenatal care, and adopt more scientifically parenting practices. These factors have a significant impact on the health of the next generation. Limited by data, this paper examines how G2 completing compulsory education impacts their own health and that of their children by improving their healthy behaviors, nutritional balance, income, and social status. In fact, there are many potential mechanisms through which an individual’s education affects the health of the subsequent generation. Some of these mechanisms are particularly important, such as individuals with better education having a higher probability of acquiring high-quality spouses and potentially adopting more scientifically informed parenting practices, which can have a significant impact on the health of the next generation. Therefore, high-quality marriages (especially marrying high-quality women) may potentially act as a significant barrier to the transmission of health disadvantage within a family. However, these mechanisms still require further examination.

Finally, this study is only a preliminary exploration of the intergenerational transmission of health in Chinese families. Our research is subject to certain limitations. For the research topic of this study, perfect data would involve measuring the health levels of grandparents, parents, and grandchildren at the same age (e.g., all at the age of 30). However, the data currently available is limited to the health status of different generations surveyed at the same time. Additionally, the health measurement used in this study is subjective self-reported health. While subjective self-reported health is strongly correlated with objective health measures [66], future research could benefit from including objective health indicators if the opportunity arises. These limitations highlight the need for further research in this area to address these crucial issues.
